# Seroprevalence of HHV-8, CMV, and EBV among the general population in Ghana, West Africa

**DOI:** 10.1186/1471-2334-8-111

**Published:** 2008-08-18

**Authors:** Andrew A Adjei, Henry B Armah, Foster Gbagbo, Isaac Boamah, Clement Adu-Gyamfi, Isaac Asare

**Affiliations:** 1 Department of Pathology, University of Ghana Medical School, College of Health Sciences, University of Ghana, Accra, Ghana; 2 Department of Microbiology, University of Ghana Medical School, College of Health Sciences, University of Ghana, Accra, Ghana

## Abstract

**Background:**

Human herpesvirus 8 (HHV-8), cytomegalovirus (CMV) and Epstein-Barr virus (EBV) are prevalent in Africa, but less common elsewhere and the modes of transmission are still subject to debate. Generally, they rarely cause disease in the immunocompetent host but are highly oncogenic when associated with immunosuppression. Although the high prevalence of HHV-8, CMV and EBV has been well documented in Africa, such data are sparse from Ghana.

**Methods:**

Serum samples from 3275 HIV-seronegative healthy blood donors and 250 HIV-AIDS patients were tested for antibodies specific for HHV-8, CMV and EBV by IgG ELISA assays. Differences in seropositivity rates by gender and age were evaluated using the Chi-square test with Yates correction.

**Results:**

Of the 3275 HIV-seronegative healthy blood donors tested, 2573 (78.6%) were males and 702 (21.4%) were females, with ages ranging from 18 to 65 years (median 32.6; mean 31.2; mode 30). Of the 250 HIV-AIDS patients tested, 140 (56%) were males and 110 (44%) were females, with ages ranging from 17 to 64 years (median 30.8; mean 30.3; mode 28). Among the HIV-seronegative healthy blood donors, overall seroprevalence of HHV-8, CMV and EBV was 23.7%, 77.6% and 20.0%, respectively. Among the HIV-AIDS patients, overall seroprevalence of HHV-8, CMV and EBV was 65.6%, 59.2% and 87.2%, respectively. The seroprevalence of HHV-8 (p < 0.005) and EBV (p < 0.001) was statistically significantly higher in HIV-AIDS patients compared to HIV-seronegative healthy blood donors. There was no statistically significant difference (p = 0.24) between CMV seroprevalence in HIV-AIDS patients and HIV-seronegative healthy blood donors. Age and gender were not independent determinants (p > 0.05) for all three infections among HIV-seronegative healthy blood donors and HIV-AIDS patients in Ghana.

**Conclusion:**

The results presented herein indicate that HHV-8, CMV and EBV infections are hyperendemic in both HIV-seronegative and HIV-seropositive Ghanaians, and suggest primarily a horizontal route of transmission of these three viral infections in Ghana.

## Background

There are currently eight known human herpesviruses: cytomegalovirus (CMV), Epstein-Barr virus (EBV), herpes simplex virus 1, herpes simplex virus 2, human herpesvirus 6, human herpesvirus 7, human herpesvirus 8 (HHV-8), and varicella-zoster virus. All eight, except herpesvirus 6 and herpesvirus 7, are known to be pathogenic to humans. HHV-8 is also known as Kaposi's sarcoma-associated herpesvirus (KSHV). HHV-8, CMV, and EBV are lymphotropic herpesviruses and responsible for a wide variety of human diseases, caused either by primary infection or by reactivation under immunosuppressive conditions. The majority (>90%) of the adult human population carries asymptomatic infection of EBV and CMV. Although HHV-8 shares substantial homology with EBV, it has a marked lower (2–30%) seroprevalence rate in the adult human population, with a specific tropism for people of Mediterranean and sub-Sahara African countries [1-4]. HHV-8 and EBV are oncogenic viruses with a long latency period in healthy hosts and will reactivate from dormancy when the hosts are immunosuppressed. Primary infections with these viruses in the immunocompetent host are generally asymptomatic. The neoplastic potentials of these two viruses have been well established, especially within the context of immunosuppressed patients who are undergoing bone-marrow transplantation or are co-infected with the human immunodeficiency virus (HIV) [5].

HHV-8 is a γ-herpesvirus that was discovered in 1994 in Kaposi's sarcoma (KS) tissues from a patient with AIDS, thereby establishing a link between HHV-8 infection and the emergence of KS. HHV-8 is now considered to be the etiological agent of all the clinico-epidemiological forms of KS (including AIDS KS, classic KS, endemic KS, and iatrogenic KS), primary effusion lymphoma, body cavity-based lymphoma, and multicentric Castleman's disease. Several studies show high prevalence rates of HHV-8 antibodies among male homosexuals, African children, Brazilian Amerindians, and elderly individuals in certain regions of the Mediterranean basin [4]. Sexual transmission of HHV-8 might play an important role among high-risk group populations, such as homosexual men in Western countries. However, in endemic areas where HHV-8 seroprevalence is high during childhood and adolescence, viral transmission might occur through nonsexual contact. This is particularly evident in African populations where high prevalence rates have been observed in infants and children, with a HHV-8 seroprevalence similar to that observed in adults [4].

CMV is a β-herpesvirus and known to be present in saliva, cervical secretions, breast milk, semen, and human lymphocytes. CMV is an ubiquitous agent, and seropositivity rates in the adult population over 40 years of age worldwide are 60 to 100%, possibly due to transmission through breastfeeding, sexual contact and spread from children [6,7]. Transfusion-transmitted CMV infection is a significant cause of morbidity and mortality, particularly in immunocompromised patients (including premature low-birth-weight infants [<1500 g] born to CMV-seronegative mothers, CMV-seronegative recipients of autologous or allogeneic bone marrow or peripheral blood stem cell transplantation, CMV-seronegative solid-organ transplant recipients, and CMV-seronegative patients with AIDS [8]. In all of these at-risk patients, it is appropriate to provide "CMV-safe" blood for transfusion.

EBV was first discovered in 1964 in Burkitt lymphoma (BL), a B-cell-derived tumor. EBV is ubiquitous in the adult population worldwide, and establishes a life-long persistent infection of B lymphocytes characterized by virus shedding into saliva [9]. African children are infected early in life and most have seroconverted by age 3 years, while in affluent countries, primary infection is delayed until young adult life [10]. EBV is now considered to be etiologically associated with endemic Burkitt's lymphoma (BL), nasopharyngeal carcinoma, classical Hodgkin's lymphoma (HL) and extranodal nasal NK/T-cell lymphoma. EBV is transmitted via saliva in an oral-fecal route of transmission, and it infects B lymphocytes as well as certain epithelial cells.

In a recent review of 28 HHV-8 seroepidemiologic studies of adult populations from 16 African countries reported between 1996 and 2002, most African countries (namely Botswana, Cameroon, Democratic Republic of Congo, Egypt, Gambia, Ghana, Ivory Coast, Nigeria, South Africa, Tanzania, Uganda, Zambia, and Zimbabwe) had high seroprevalence rates ranging from 26% to 86%, with the exception of Central African Republic, Eritrea and Senegal which had relatively lower (up to 25%) seroprevalence rates [11]. Another recent review of 7 HHV-8 seroepidemiologic studies of pediatric populations from 7 African countries reported between 1998 and 2003 showed high seroprevalence rates ranging from 30% to 58.1% in most African countries (namely Cameroon, Egypt, Ghana, Tanzania, Uganda, and Zambia), with the exception of Eritrea which had a very low (up to 2%) seroprevalence rate [4]. Although Kaposi's sarcoma is common in Ghana compared to other cutaneous malignancies [12], data on the seroprevalence of HHV-8 in Ghana are scanty. Ablashi and colleagues first reported a HHV-8 seroprevalence rate of 41.9% in healthy Ghanaians aged 13–72 years in 1999 [3]. Subsequently, Nuvor and colleagues reported statistically significant (p < 0.05) difference in HHV-8 seroprevalence rate between HIV-seronegative healthy blood donors (32.3%) and asymptomatic HIV-seropositive individuals (45.5%) in Ghana [13]. Data on the prevalence of CMV and EBV infections in Ghana are even scantier. The reported seroprevalence rate of CMV among healthy Ghanaian blood donors aged 18–60 years was 93.2% [14]. EBV viral DNA was detected in plasma samples of 40% (47% in the malaria-infected and 34% in the non-malaria group) of Ghanaian children aged 6 months to 12 years [15]. The aim of this study was to determine and compare the seroprevalence of HHV-8, CMV, and EBV infections among HIV-AIDS patients and HIV-seronegative healthy blood donors in Ghana, in an effort to further define the seroepidemiology and transmission of these infections in Ghana.

## Methods

### Study sites and populations

Three thousand two hundred and seventy-five HIV-seronegative serum samples were obtained from 7 of the 10 regional blood banks of the Ghana National Blood Transfusion Service from healthy blood donors who gave their informed consent between 2001 and 2002. In Ghana, blood donors are volunteers (both occasional and periodic repeat non-compensated volunteer donors) and are also sought from family members and friends of patients requiring blood transfusion. They are selected based on the following criteria: age between 18 and 65 years; weight >45 kg; haemoglobin >12.5 g/dl; normal blood pressure [BP], pulse, and body temperature; and not belonging to any high-risk group (homosexually or heterosexually promiscuous, intravenous drug users; history of sexually transmitted diseases; and history of any severe current or chronic illnesses). Donated blood is routinely screened for HIV 1 & 2, HBsAg, anti-HCV and syphilis antibodies in Ghana.

Two hundred and fifty HIV-AIDS patients with chronic diarrhea on admission to the Fevers Unit of the Korle-Bu Teaching Hospital, Accra, Ghana, were recruited for the study between February 2001 and June 2002. The Korle-Bu Teaching Hospital is the leading tertiary hospital in Ghana that serves the city of Accra, its surrounding urban population, and the southern part of Ghana. Accra, the capital city of Ghana, is a rapidly expanding city with a population of approximately 3 million. All 250 HIV-AIDS patients (with a single infection from HIV-1) fell within the Centers for Disease Control and Prevention (CDC) clinical staging A1–C3 categories, representing asymptomatic to severe AIDS conditions. The participating HIV-AIDS patients had mean CD4 counts of 288 cells per microliter (95% confidence interval of 237–340 cells per microliter). All participating HIV-AIDS patients reported watery stools lasting from 3–90 days (diarrhea episodes, 3–10 watery stools per day). The HIV testing for antibodies to HIV-1 and HIV-2 in the study HIV-AIDS patients and blood donors was done by the particle agglutination test (Serodia HIV-1 and HIV-2; Serodia Fujirebio Inc., Tokyo, Japan) and confirmed by Western blot analysis (New Lav Blot I and II; Sanofi Diagnostic Pasteur, Marnes-la-Coquette, France). The study received ethical review and approval from the Ethical and Protocol Review Committee of the University of Ghana Medical School, Accra, Ghana, and written informed consent was obtained from all study participants.

### Serological analysis

Sera were tested at the Virology Unit, Noguchi Memorial Institute for Medical Research, for the presence of antibodies to HHV-8 (ELISA; IgG; Advanced Biotechnologies, Columbia, Maryland, USA), antibodies to CMV (ELISA; IgG; Diamedix Corporation, Miami, Florida, USA), and antibodies to EBV (ELISA; IgG antibody to viral capsid antigen; Advanced Biotechnologies, Columbia, Maryland, USA), in accordance with the respective manufacturer's instructions. Positive and negative standard sera, accompanying the kit were included in each assay.

### Statistical analysis

The Statistical Analysis System (SAS Institute, Cary, NC, USA) version 9.1 was used to complete all data analyses. Seropositivity rates were calculated and compared by gender and among different 10-year interval age groups. Differences were evaluated using the Chi-square test with Yates correction. A P value of < 0.05 was considered statistically significant.

## Results

Of the 3275 HIV-seronegative healthy blood donors tested, 2573 (78.6%) were males and 702 (21.4%) were females, with ages ranging from 18 to 65 years (median 32.6; mean 31.2; mode 30). Of the 250 HIV-AIDS patients tested, 140 (56%) were males and 110 (44%) were females, with ages ranging from 17 to 64 years (median 30.8; mean 30.3; mode 28).

Table 1 shows HHV-8, CMV and EBV seropositivity according to age and gender among the 3275 HIV-seronegative healthy blood donors in Ghana. Among the 3275 HIV-seronegative healthy blood donors, overall seroprevalence of HHV-8, CMV and EBV was 23.7%, 77.6% and 20.0%, respectively (Table 1). There was no statistically significant (p > 0.05) difference in the overall seroprevalence of HHV-8 between male and female HIV-seronegative healthy blood donors. Additionally, there was no statistically significant (p > 0.05) difference in the overall seroprevalence of CMV between male and female HIV-seronegative healthy blood donors. Finally, there was no statistically significant (p > 0.05) difference in the overall seroprevalence of EBV between male and female HIV-seronegative healthy blood donors [Table 1]. Hence, gender was not an independent determinant (p > 0.05) for all three infections among HIV-seronegative healthy blood donors in Ghana.

**Table 1:** HHV-8, CMV and EBV seropositivity according to age and gender among the 3275 HIV-seronegative healthy blood donors in Ghana

Age group, years	Proportion (%) of HIV-seronegative blood donors with HHV8 seropositivity			Proportion (%) of HIV-seronegative blood donors with CMV seropositivity			Proportion (%) of HIV-seronegative blood donors with EBV seropositivity		
	Male	Female	All	Male	Female	All	Male	Female	All

16–25	104/681 (15.3)	65/335 (19.4)	169/1016 (16.6)	558/681 (81.9)	232/335 (69.3)	790/1016 (77.8)	108/681 (15.9)	74/335 (22.1)	182/1016 (17.9)
26–35	241/1120 (21.5)	88/220 (40.0)	329/1340 (24.6)	831/1120 (74.2)	158/220 (71.8)	989/1340 (73.8)	35/1120 (3.1)	28/220 (12.7)	63/1340 (4.7)
36–45	118/478 (24.7)	29/74 (39.2)	147/552 (26.6)	423/478 (88.5)	57/74 (77.0)	480/552 (87.0)	223/478 (46.7)	36/74 (48.6)	259/552 (46.9)
46–55	48/158 (30.4)	12/42 (28.6)	60/200 (30.0)	101/158 (63.9)	37/42 (88.1)	138/200 (69.0)	76/158 (48.1)	18/42 (42.9)	94/200 (47.0)
56–65	56/136 (41.2)	16/31 (51.6)	72/167 (43.1)	120/136 (88.2)	25/31 (80.6)	145/167 (86.8)	40/136 (29.4)	18/31 (58.1)	58/167 (34.7)
Total	567/2573 (22.0)	210/702 (29.9)	777/3275 (23.7)	2033/2573 (79.0)	509/702 (72.5)	2542/3275 (77.6)	482/2573 (18.7)	174/702 (24.8)	656/3275 (20.0)

Table 2 shows HHV-8, CMV and EBV seropositivity according to age and gender among the 250 HIV-AIDS patients in Ghana. Among the 250 HIV-AIDS patients, overall seroprevalence of HHV-8, CMV and EBV was 65.6%, 59.2% and 87.2%, respectively (Table 2). There was no statistically significant (p > 0.05) difference in the overall seroprevalence of HHV-8 between male and female HIV-AIDS patients. Additionally, there was no statistically significant (p > 0.05) difference in the overall seroprevalence of CMV between male and HIV-AIDS patients. Finally, there was no statistically significant (p > 0.05) difference in the overall seroprevalence of EBV between male and HIV-AIDS patients [Table 2]. Hence, gender was not an independent determinant (p > 0.05) for all three infections among HIV-AIDS patients in Ghana.

**Table 2:** HHV-8, CMV and EBV seropositivity according to age and gender among the 250 HIV-AIDS patients in Ghana

Age group, years	Proportion (%) of HIV-AIDS patients with HHV8 seropositivity			Proportion (%) of HIV-AIDS patients with CMV seropositivity			Proportion (%) of HIV-AIDS patients with EBV seropositivity		
	Men	Women	All	Men	Women	All	Men	Women	All

16–25	11/18 (61.1)	13/19 (68.4)	24/37 (64.9)	12/18 (66.7)	10/19 (52.6)	22/37 (59.5)	16/18 (88.9)	14/19 (73.7)	30/37 (81.1)
26–35	36/49 (73.5)	29/41 (70.7)	65/90 (72.2)	31/49 (63.3)	28/41 (68.3)	59/90 (65.6)	45/49 (91.8)	35/41 (85.4)	80/90 (88.9)
36–45	24/38 (63.2)	21/35 (60.0)	45/73 (61.6)	22/38 (57.9)	19/35 (54.3)	41/73 (56.2)	35/38 (92.1)	30/35 (85.7)	65/73 (89.0)
46–55	12/20 (60.0)	9/16(56.3)	21/36 (58.3)	10/20 (50.0)	8/16 (50.0)	18/36 (50.0)	17/20 (85.0)	14/16 (87.5)	31/36 (86.1)
56–65	5/8 (62.5)	4/6 (66.7)	9/14 (64.3)	5/8 (62.5)	3/6 (50.0)	8/14 (57.1)	7/8 (87.5)	5/6 (83.3)	12/14 (85.7)
Total	88/140 (62.9)	76/110 (69.1)	164/250 (65.6)	80/140 (57.1)	68/110 (61.8)	148/250 (59.2)	122/140 (87.1)	96/110 (87.3)	218/250 (87.2)

Table 3 shows the comparison of seroprevalence of HHV-8, CMV and EBV between HIV-seronegative healthy blood donors and HIV-AIDS patients in Ghana by age and gender. The overall seroprevalence of HHV-8 was statistically significantly (p < 0.005) higher in HIV-AIDS patients (65.6%, 164/250) compared to HIV-seronegative healthy blood donors (23.7%, 777/3275). Additionally, the seroprevalence of HHV-8 was statistically significantly (p < 0.005) higher in male HIV-AIDS patients (62.9%, 88/140) compared to male HIV-seronegative healthy blood donors (22.0%, 567/2573). Furthermore, the seroprevalence of HHV-8 was statistically significantly (p < 0.05) higher in female HIV-AIDS patients (69.1%, 76/110) compared to female HIV seronegative healthy blood donors (29.9%, 210/702). Finally, the seroprevalence of HHV-8 was statistically significantly (p < 0.05) higher in HIV-AIDS patients compared to HIV-seronegative healthy blood donors within each of the five 10-year interval age groups (Table 3).

**Table 3:** Comparison of seroprevalence of HHV-8, CMV and EBV between HIV-seronegative healthy blood donors and HIV-AIDS patients in Ghana by age and gender

Patient characteristic	Proportion (%) of HHV-8 seropositives			Proportion (%) of CMV seropositives			Proportion (%) of EBV seropositives		
	HIV-	HIV+	*P value	HIV-	HIV+	*P value	HIV-	HIV+	*P value

Age group, years									
16–25	169/1016 (16.6)	24/37 (64.9)	< 0.001	790/1016 (77.8)	22/37 (59.5)	0.26	182/1016 (17.9)	30/37 (81.1)	< 0.0005
26–35	329/1340 (24.6)	65/90 (72.2)	< 0.005	989/1340 (73.8)	59/90 (65.6)	0.45	63/1340 (4.7)	80/90 (88.9)	< 0.0001
36–45	147/552 (26.6)	45/73 (61.6)	< 0.05	480/552 (87.0)	41/73 (56.2)	0.10	259/552 (46.9)	65/73 (89.0)	< 0.05
46–55	60/200 (30.0)	21/36 (58.3)	< 0.05	138/200 (69.0)	18/36 (50.0)	0.25	94/200 (47.0)	31/36 (86.1)	< 0.05
56–65	72/167 (43.1)	9/14 (64.3)	< 0.05	145/167 (86.8)	8/14 (57.1)	0.12	58/167 (34.7)	12/14 (85.7)	< 0.05
Gender									
Male	567/2573 (22.0)	88/140 (62.9)	< 0.005	2033/2573 (79.0)	80/140 (57.1)	0.19	482/2573 (18.7)	122/140 (87.1)	< 0.0005
Female	210/702 (29.9)	76/110 (69.1)	< 0.05	509/702 (72.5)	68/110 (61.8)	0.36	174/702 (24.8)	96/110 (87.3)	< 0.005
Total	777/3275 (23.7)	164/250 (65.6)	< 0.005	2542/3275 (77.6)	148/250 (59.2)	0.24	656/3275 (20.0)	218/250 (87.2)	< 0.001

*Comparison between HIV-seronegative blood donors and HIV-AIDS patients using the chi-square test with Yates correction.

There was no statistically significant (p = 0.24) difference in the overall seroprevalence of CMV between HIV-AIDS patients and HIV-seronegative healthy blood donors. Additionally, the seroprevalence of CMV was not statistically significantly (p = 0.19) different between male HIV-AIDS patients and male HIV-seronegative healthy blood donors. Furthermore, the seroprevalence of CMV was not statistically significantly (p = 0.36) different between female HIV-AIDS patients and female HIV seronegative healthy blood donors. Finally, the seroprevalence of CMV was not statistically significantly (p > 0.05) different between HIV-AIDS patients and HIV-seronegative healthy blood donors within each of the five 10-year interval age groups (Table 3).

The overall seroprevalence of EBV was statistically significantly (p < 0.001) higher in HIV-AIDS patients (87.2%, 218/250) compared to HIV-seronegative healthy blood donors (20.0%, 656/3275) [Table 3]. Additionally, the seroprevalence of EBV was statistically significantly (p < 0.0005) higher in male HIV-AIDS patients (87.1%, 122/140) compared to male HIV-seronegative healthy blood donors (18.7%, 482/2573). Furthermore, the seroprevalence of EBV was statistically significantly (p < 0.005) higher in female HIV-AIDS patients (87.3%, 96/110) compared to female HIV seronegative healthy blood donors (24.8%, 174/702). Finally, the seroprevalence of EBV was statistically significantly (p < 0.05) higher in HIV-AIDS patients compared to HIV-seronegative healthy blood donors within each of the five 10-year interval age groups (Table 3).

The seroprevalence of HHV-8 among HIV-seronegative healthy blood donors increased with increasing age; with lowest (16.6%) in 16–25 age group, through 24.6% in 26–35 age group, 26.6% in 36–45 age group, 30.0% in 46–55 age group, and highest (43.1%) in 56–65 age group (Tables 1 &3). However, the increasing HHV-8 seropositivity among HIV-seronegative healthy blood donors with increasing age did not reach the level of statistical significance (p for trend > 0.05, data not shown). The seroprevalence of HHV-8 among HIV-AIDS patients was lowest (58.3%) in 46–55 age group and highest (72.2%) in 26–35 age group (Tables 2 &3), and there was no statistically significant difference in HHV-8 seropositivity among HIV-AIDS patients between the different age groups (p for trend > 0.05, data not shown). The seroprevalence of CMV among HIV-seronegative healthy blood donors was lowest (69.0%) in 46–55 age group and highest (87.0%) in 36–45 age group (Tables 1 &3), and there was no statistically significant difference in CMV seropositivity among HIV-seronegative healthy blood donors between the different age groups (p for trend > 0.05, data not shown). The seroprevalence of CMV among HIV-AIDS patients was lowest (50.0%) in 46–55 age group and highest (65.6%) in 26–35 age group (Tables 2 &3), and there was no statistically significant difference in CMV seropositivity among HIV-AIDS patients between the different age groups (p for trend > 0.05, data not shown). The seroprevalence of EBV among HIV-seronegative healthy blood donors was lowest (4.7%) in 26–35 age group and highest (47.0%) in 46–55 age group (Tables 1 &3), and there was no statistically significant difference in EBV seropositivity among HIV-seronegative healthy blood donors between the different age groups (p for trend > 0.05, data not shown). The seroprevalence of EBV among HIV-AIDS patients was lowest (81.1%) in 16–25 age group and highest (89.0%) in 36–45 age group (Tables 2 &3), and there was no statistically significant difference in EBV seropositivity among HIV-AIDS patients between the different age groups (p for trend > 0.05, data not shown). Hence, age was not an independent determinant (p > 0.05) for all three infections among both HIV-seronegative healthy blood donors and HIV-AIDS patients in Ghana.

## Discussion

Several studies have suggested that HHV-8 transmission may differ between endemic and non-endemic countries. In countries where infection is highly endemic, HHV-8 seroprevalence is very low in children under 2 years of age and increases soon after that age [16-19]. These seroepidemiologic studies suggest that HHV-8 is mainly transmitted among family members and close contacts via a horizontal, non-sexual route; transmission during pregnancy and through breastfeeding having a minimal role in propagating the virus [16-21]. Other studies have suggested that sexual transmission also occurs in endemic populations [17,22-24]. Volpi and colleagues [23] recently demonstrated a statistically significant association between HHV-8 and HSV-2 (a prototypic sexually transmitted infection) in Northern Cameroon (a HHV-8 endemic African country), thus suggesting sexual transmission of these two viruses with HSV-2 probably facilitating the sexual transmission of HHV-8 infection in endemic countries. Additionally, Rezza and colleagues [24] demonstrated a statistically significant association between HIV, HHV-8 and EBV in Northern Cameroon, thus suggesting their shared mode of transmission. In non-endemic countries, heterosexual transmission is probably not frequent [25]. In contrast, sexual transmission is more common among men who have sex with men in non-endemic countries [18]. Several studies have demonstrated that saliva is the principal reservoir for HHV-8, whereas the viral load of HHV-8 is consistently lower in peripheral blood, secretions from genital sites, and semen [17,18,26,27]. Therefore, although HHV-8 may be transmitted mainly through saliva in endemic countries like Ghana and Cameroon, sexual transmission may be an important additional mode of transmission in endemic African population [17,22-24].

The herein reported high seroprevalence of HHV-8 in both HIV-seronegative healthy blood donors (23.7%) and HIV-AIDS patients (65.6%) confirms that HHV-8 is endemic in Ghana, and is consistent with the range of 32.3–45.5% previously reported in Ghana [3,13]. Additionally, our finding confirms the known endemicity of HHV-8 in the general population of African countries [4,11]. However, the herein reported HHV-8 seroprevalence rate of 23.7% among Ghanaian healthy blood donors is higher than the recently reported HHV-8 seroprevalence rate of 11.5% among blood donors in Burkina Faso [28], the immediate northern neighbour of Ghana. The herein reported prevalence rate of 77.6% for CMV IgG among HIV-seronegative healthy blood donors is comparable to the rate (93.2% for CMV IgG) recently reported among a smaller sample of HIV-seronegative healthy blood donors at one blood bank in Ghana [14]. The high CMV seropositivity rate in Ghana is suggestive of ubiquitous past exposure to infection. The high CMV seropositivity rate in blood donors reported in this study is comparable to the rates reported in Tunisia (97.0%) [29] and India (96.0%) [30], respectively. Additionally, the hyperendemicity of CMV in Ghana may explain the herein reported lack of statistically significant differences in the seroprevalence of CMV in HIV-seronegative healthy blood donors and HIV-AIDS patients between the sexes and the different age groups.

The herein reported significantly higher seroprevalence of HHV-8 and EBV in HIV-AIDS patients compared to HIV-seronegative healthy blood donors suggests that sexual transmission might play an important role among high-risk sexual behaviour populations, such as HIV-seropositive individuals. The herein significantly higher seroprevalence of HHV-8 in HIV-AIDS patients compared to HIV-seronegative healthy blood donors is consistent with one previous study in Ghana, which reported statistically significant (p < 0.05) difference in HHV-8 seroprevalence rate between HIV-seronegative healthy blood donors (32.3%) and asymptomatic HIV-seropositive individuals (45.5%) [13]. However, the herein reported comparably high seroprevalence of HHV-8, CMV and EBV during both adolescence and adulthood suggests that their transmission might occur primarily through horizontal, non-sexual, contact. Indeed, this is particularly evident in African populations where high prevalence rates have been observed in infants and children, with seroprevalence rates similar to that observed in adults [4,11,20,21]. This large seroepidemiology study supports the view that these three viral infections are primarily transmitted non-sexually in Ghana. Therefore, non-sexual transmission mainly through close interpersonal (especially between mother and child and among siblings) contact of non-intact skin or mucous membranes with blood containing secretions or saliva, may be the primary mode of transmission of HHV-8 in Ghana, similar to that suggested in previous reports from endemic areas [4,11,20,21]. However, the relatively smaller number of HIV-AIDS patients compared to HIV-seronegative healthy blood donors in this study may be a limitation. Therefore, we suggest that further epidemiological studies should be carried out in Ghana in order to understand the relationship between HIV and HHV-8 infection in association with KS among the general population and HIV-infected individuals.

An important issue that has major public health implications is the possibility of transmission of HHV-8, CMV and EBV through blood transfusion [7,8,31,32], especially in hyperendemic countries such as Ghana. Of these three viruses, cytomegalovirus (CMV) is the only one that has assumed very significant importance in blood transfusion [32]. The American Association of Blood Banks recommends the transfusion of "CMV-safe" (CMV-seronegative or leukocyte-reduced) blood to at-risk individuals, and this has been the standard of care in most developed countries since the late 1980s. These guidelines have helped in drastically minimizing transfusion-transmitted CMV infection in immunosuppressed recipients [32-34]. The observed high seroprevalence of CMV among Ghanaian blood donors does not justify pre-donation blood donor screening for this virus in Ghana because CMV serology is just a proxy of viremia, blood donor screening for CMV would be an obstacle to blood supply in Ghana, and CMV-seronegative blood is recommended only for organ recipients or other immunosuppressed patients. However, it does justify post-donation testing of donated blood in Ghana for CMV in order to identify the very few CMV-seronegative blood donors, motivate these CMV-seronegatives to become periodic repeat non-compensated volunteer donors, maintain a database of the epidemiological and contact information of these CMV-seronegatives to enable their rapid recall in times of need, and educate and counsel these CMV-seronegatives on how to maintain their status and the importance of their status for themselves and the increasing immunosuppressed population in Ghana. Additionally, the above proposed post-donation testing of donated blood for CMV and the subsequent determination of the actual titres of neutralization antibodies in the numerous CMV-seropositives will ensure the identification of those CMV-seropositives with very high neutralizing antibody titres from whom immunoglobulins can be obtained to treat CMV infections in at-risk individuals, and who will be followed-up and recalled when necessary in the same manner described above for the few CMV-seronegatives. However, the maintenance of CMV-seropositive and CMV-seronegative "dual inventories" in blood banks is expensive, and some countries with high CMV seroprevalence have found it difficult to maintain adequate supplies of CMV-seronegative products [35], as would be the case for a developing country such as Ghana. Thus, alternate methods for the provision of "CMV safe" blood products have been pursued, including the use of leukocyte-reduced blood products. The question whether the use of CMV-seronegative versus leukocyte-reduced blood components is equally efficacious in preventing transfusion-acquired CMV infection remains unresolved in the literature [36,37]. Bowden and colleagues reported that the use of leukocyte-reduced blood products was comparable to the use of CMV-seronegative blood products for the prevention of transfusion-transmitted CMV infection after marrow transplant [36]. However, a recent meta-analysis of the available controlled studies indicated that CMV-seronegative blood components were more efficacious than leukocyte-reduced blood components in preventing transfusion-acquired CMV infection [37].

## Conclusion

The high seroprevalence of the three viruses among both HIV-positive and HIV-negative individuals suggests endemicity and predominant horizontal, non-sexual, transmission of the infections in Ghana. The higher seroprevalence of HHV-8 and EBV among HIV-AIDS patients compared to healthy blood donors suggests an additional role of sexual transmission.

## Abbreviations

AIDS: acquired immunodeficiency syndrome; CMV: cytomegalovirus; EBV: Epstein Barr virus; HHV-8: human herpes virus 8; HIV: human immunodeficiency virus; KSHV: Kaposi's sarcoma-associated herpesvirus; OR: odds ratio; 95% CI: 95% confidence interval

## Competing interests

The authors declare that they have no competing interests.

## Authors' contributions

AAA conceived study, provided guidance to all aspects of study, and revised manuscript for important intellectual content. HBA performed quality assessment of data, data analysis, data preparation, and drafted manuscript. AAA, HBA, FG, IB, CA and IA participated in design and coordination of study, data and sample collection, and performed and supervised immunoassays. All authors read and approved final manuscript.

## Pre-publication history

The pre-publication history for this paper can be accessed here:


